# AuPt bimetallic nanocatalyst-amplified luminol-dissolved oxygen electrochemiluminescence for interferon-γ immunosensing

**DOI:** 10.3389/fchem.2026.1942955

**Published:** 2026-09-03

**Authors:** Huanrui Wang, Jiaxuan Li, Liang Cui

**Affiliations:** 1 Peking University People’s Hospital, Beijing, China; 2 The Institute of Applied Lithotripsy Technology, Peking University, Beijing, China; 3 School of Chemistry and Chemical Engineering, Zhejiang Sci-Tech University, Hangzhou, China

**Keywords:** AuPt bimetallic nanoparticles, electrochemiluminescence, immunosensor, interferon-γ, luminol-dissolved oxygen

## Abstract

Interferon-γ (IFN-γ) is a pivotal inflammatory cytokine whose abnormal expression is associated with autoimmune disorders, infectious diseases, and cancer. Herein, a signal-off electrochemiluminescence (ECL) immunosensor was developed by integrating catalytic signal amplification with oriented antibody immobilization. An amino-functionalized vertically ordered mesoporous silica film (NH_2_-VMSF) was rapidly grown on indium tin oxide (ITO) electrode by electrochemically assisted self-assembly (EASA), and gold-platinum bimetallic nanoparticles (AuPt NPs) were electrodeposited under nanoconfinement within its vertical nanochannels. The coupled Au and Pt sites promoted both dissolved oxygen (DO) reduction to superoxide radical anion (O_2_^•−^) and luminol oxidation, thereby enhancing luminol-DO ECL response. For construction of the biorecognition interface, glutaraldehyde was attached to the external film surface of NH_2_-VMSF while surfactant micelles still occupied the nanochannels, minimizing nanochannel blockage. Protein A was subsequently covalently coupled and used to orient anti-IFN-γ antibodies through their fragment crystallizable regions. IFN-γ binding formed an interfacial immunocomplex layer that hindered the transport of luminol, producing a concentration-dependent decrease in ECL intensity. The immunosensor can detect IFN-γ from 100 fg mL^−1^–100 ng mL^−1^ with limit of detection (LOD) of 31 fg mL^−1^. This platform provides sensitive strategy for cytokine analysis in complex biological matrices using catalytically amplified luminol-DO ECL system.

## 1 Introduction

As a key proinflammatory cytokine, interferon-γ (IFN-γ) plays central roles in host immune defense, immune surveillance, and inflammatory regulation (Dunn et al., 2006). Its expression level can provide valuable information for evaluating immune status and supporting disease assessment (e.g. autoimmune, infectious, and neoplastic diseases, including rheumatoid arthritis, tuberculosis, acquired immunodeficiency syndrome, melanoma, and several other cancers) (Sánchez-Tirado et al., 2020; Yeh et al., 2023; Zhou Y. et al., 2022). However, physiological IFN-γ concentrations in human serum are generally at the pg mL^−1^ level (Yerrapragada and Mampallil, 2022), and the complex serum matrix can interfere with analytical measurements. Rapid, sensitive, and accurate determination of IFN-γ is important for early disease screening, disease monitoring, and therapeutic evaluation.

Currently available IFN-γ assays mainly rely on immunochemical methods. The immobilization of antibodies on solid substrates is a critical factor affecting immunosensor performance. Conventional random immobilization may produce disordered antibody orientations and can involve the antigen-binding domains in the coupling reaction, thereby reducing recognition activity. An antibody contains two fragment antigen-binding (Fab) arms and one fragment crystallizable (Fc) region, while the Fc region does not directly involve in antigen recognition (Lu et al., 1996). As conventional covalent coupling cannot distinguish between these functional regions, immobilization through Fab-associated residues may partially or completely impair antigen binding. Several oriented immobilization strategies have been developed using protein A (PrA) or protein G that bind the antibody Fc region with high affinity (Fang et al., 2026). For instance, PrA is attractive as an intermediate layer because it binds the Fc regions of antibody, leaving the Fab domains more accessible to target antigens.

According to the signal readout mode, IFN-γ immunosensing includes enzyme-linked immunosorbent assay (ELISA) (Le and quin, 2005), immunofluorescence, surface plasmon resonance (Stybayeva et al., 2010), and IFN-γ release assays based on ELISA or enzyme-linked immunospot testing (Wang et al., 2013). Although these methods have been widely used, they also suffer from long assay times, expensive instruments, interference from background signals, or insufficient sensitivity. Electrochemiluminescence (ECL) sensors recently have attracted considerable attention because of their low optical background, broad dynamic range, and operational simplicity (Huang et al., 2024a; Li J. et al., 2026; Luo et al., 2022; Shi et al., 2025). Luminol is a widely employed ECL emitter, offering advantages of low cost, low toxicity, and low onset potential for light emission (Lin et al., 2026a; Lin et al., 2026b; Zhang et al., 2026). However, its intrinsic ECL intensity is generally weak, making the introduction of a co-reactant necessary for signal amplification. Hydrogen peroxide (H_2_O_2_) is frequently used for this purpose (Cui et al., 2026; Huang et al., 2026; Wu et al., 2025; Yan et al., 2026), but its limited chemical stability and strong oxidizing ability might adversely affect biomolecular activity. In contrast, dissolved oxygen (DO) is non-toxic, stable, and naturally available in aqueous electrolyte solutions. However, its activation kinetics are slow under the neutral or mildly alkaline conditions required to preserve biomolecular activity. Catalytic acceleration of DO reduction is therefore an effective route for enhancing the ECL signal (Fan et al., 2025a; Jiang et al., 2026; Zhang et al., 2025; Zhao et al., 2025a).

Noble-metal nanomaterials have recently been used to catalyze electrochemical oxygen reduction and promote luminol ECL (Pei et al., 2025; Zhou et al., 2025). Both gold nanoparticles and platinum nanoparticles can catalyze oxygen reduction reaction (ORR) to reactive oxygen species (ROS), thereby enhancing luminol-DO ECL (An et al., 2024; Li et al., 2024). Combining Au and Pt within a bimetallic nanostructure can further improve catalytic activity through electronic coupling and complementary catalytic functions while reducing the amount of either individual noble metal (Wang and Li, 2011). However, the controllable preparation of bimetallic nanoparticles remains challenging because of differences in the intrinsic physicochemical properties of the constituent metals, thermodynamic instability, and particle aggregation (Yang et al., 2020).

Vertically ordered mesoporous silica film (VMSF) consists of uniformly sized nanochannels aligned perpendicular to the supporting substrate (Ma et al., 2024; Yang et al., 2022; Yu et al., 2024; Zhao et al., 2026). Their pore diameters are typically approximately 2–3 nm (Deng et al., 2023; Huang et al., 2022; Huang J. et al., 2023). VMSF possesses high specific surface area, chemical stability, convenient surface functionalization, charge-selective permeability, and efficient molecular transport (Gu et al., 2026; Huang et al., 2024b; Su et al., 2022; Wu et al., 2026a; Zhou H. et al., 2022). When these nanochannels are used as confined templates for metal deposition, they can restrict nanoparticle growth, inhibit aggregation, and improve the spatial distribution and utilization of catalytic sites (Fan et al., 2024; Huang L. et al., 2023; Lu et al., 2026a). VMSF therefore provides an attractive platform for constructing confined metallic nanostructures and high-performance ECL sensing interfaces (Wu L. et al., 2026).

In this work, an amino-functionalized VMSF (NH_2_-VMSF) was grown on ITO electrode, and gold-platinum bimetallic nanoparticles (AuPt NPs) were confined within the vertical nanochannels by electrodeposition. The AuPt NPs promoted both dissolved-oxygen reduction and electrochemical oxidation of luminol, significantly amplifying the ECL response. Glutaraldehyde-mediated coupling of PrA to the external NH_2_-VMSF surface enabled Fc-directed immobilization of anti-IFN-γ antibodies. Binding of IFN-γ increased interfacial steric hindrance and suppressed the transport of luminol to the confined catalytic sites and the underlying electrode. This signal-off mechanism enabled sensitive determination of IFN-γ.

## 2 Materials and methods

The reagent, material, characterization, methods for electrode modification with NH_2_-VMSF or AuPt NPs were provided in Supplementary Material.

### 2.1 Construction of the ECL immunosensor

Glutaraldehyde (GA) functionalization was performed before removal of CTAB micelles (SM@NH_2_-VMSF/ITO) to introduce reactive aldehyde groups on external NH_2_-VMSF surface while minimizing GA entry into the nanochannels. Briefly, SM@NH_2_-VMSF/ITO electrode was immersed in 5 wt% GA solution and incubated in dark at 37 °C for 30 min followed with rinsing with phosphate buffer solution (PBS, 0.01 M, pH 7.4). The GA-functionalized electrode was subsequently immersed in 0.1 M HCl/ethanol for 5 min to remove CTAB micelles, obtaining GA/NH_2_-VMSF/ITO electrode. Then, AuPt NPs were co-electrodeposited within NH_2_-VMSF nanochannels using constant-potential method. The deposition solution contained 0.25 mM gold (III) chloride and 0.75 mM hexachloroplatinic acid in 0.1 M sulfuric acid. Electrodeposition was performed on NH_2_-VMSF/ITO at −0.2 V for 1 s, yielding GA/AuPt NPs@NH_2_-VMSF/ITO electrode. Then, the electrode was incubated with 100 μg mL^−1^ PrA at 4 °C for 60 min following with carefully rinse with 0.01 M PBS at pH 7.4. A 10 μg mL^−1^ anti-IFN-γ antibody was then applied and incubated at 4 °C for 60 min to allow Fc-directed antibody (Ab) immobilization through PrA. Unbound antibodies were removed by rinsing with PBS. Finally, 0.1% bovine serum albumin (BSA) solution was applied to block residual non-specific binding sites. After rinsing and drying, the ECL immunosensor, denoted BSA/Ab/PrA/GA/AuPt NPs@NH_2_-VMSF/ITO, was obtained.

### 2.2 ECL detection of IFN-γ

For IFN-γ determination, the immunosensor was incubated with known concentration of IFN-γ at 4 °C for 90 min, allowing sufficient binding between IFN-γ and the surface-immobilized Ab. After incubation, the electrode was gently rinsed with 0.01 M PBS at pH 7.4. ECL signals were recorded using 0.01 M PBS at pH 7.4 containing 100 μM luminol at a photomultiplier tube (PMT) bias of 600 V. The ECL response was excited by cyclic voltammetry (CV) scanning (−1.0 to 0.8 V at 100 mV s^−1^). All analytical measurements were performed in air-equilibrated solution under ambient atmospheric conditions to ensure stable DO concentration. For matrix-spiked recovery analysis, commercial FBS was spiked with IFN-γ standard solutions. Each spiked FBS sample was diluted 50-fold with PBS (0.01 M, pH 7.4) before incubation with the immunosensor. The IFN-γ concentration in the diluted sample was detected. Recovery was calculated using found concentration (*C*_found_) and added concentration (*C*_added_) as *C*_found_/C_added_ × 100%. Each measurement was performed in triplicate.

## 3 Results and discussion

### 3.1 Design of immunosensor and sensing principle

**FIGURE 1:**
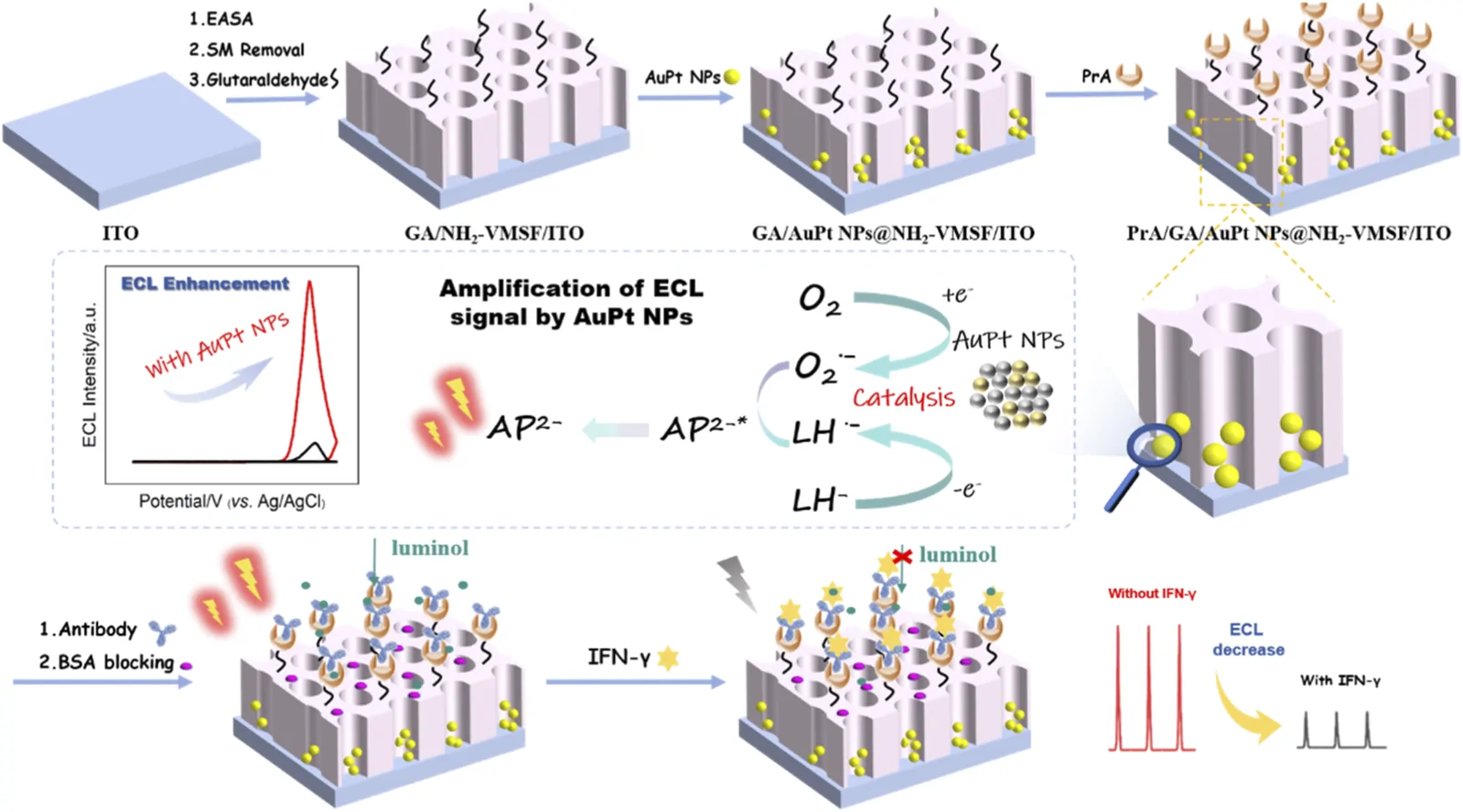
Schematic illustration of fabrication of immunosensor and the proposed ECL detection using AuPt NPs enhanced luminol-DO ECL and Fc-directed immobilization of Ab through PrA.

The immunosensor was constructed by combining nanochannel-confined AuPt catalysis with PrA-mediated oriented antibody immobilization (Figure 1). NH_2_-VMSF was first grown on ITO surface using simple EASA method (Gong et al., 2022a; Gong et al., 2022b; Zhou et al., 2024), after which AuPt NPs were electrodeposited within vertical nanochannels. The coupled Au and Pt sites promoted dissolved-oxygen reduction to ROS and facilitated luminol oxidation, thereby amplifying luminol-DO ECL response. For construction of the recognition interface, PrA was covalently immobilized on external surface of NH_2_-VMSF through GA. The specific interaction between PrA and the antibody Fc region produced a more favorable antibody orientation and improved the accessibility of the antigen-binding sites. Binding of IFN-γ to the immobilized antibody generated an insulating and sterically hindered immunocomplex layer. This layer suppressed the transport of luminol to AuPt catalytic sites within the nanochannels, leading to concentration-dependent ECL decrease.

### 3.2 Material and electrode characterization

**FIGURE 2:**
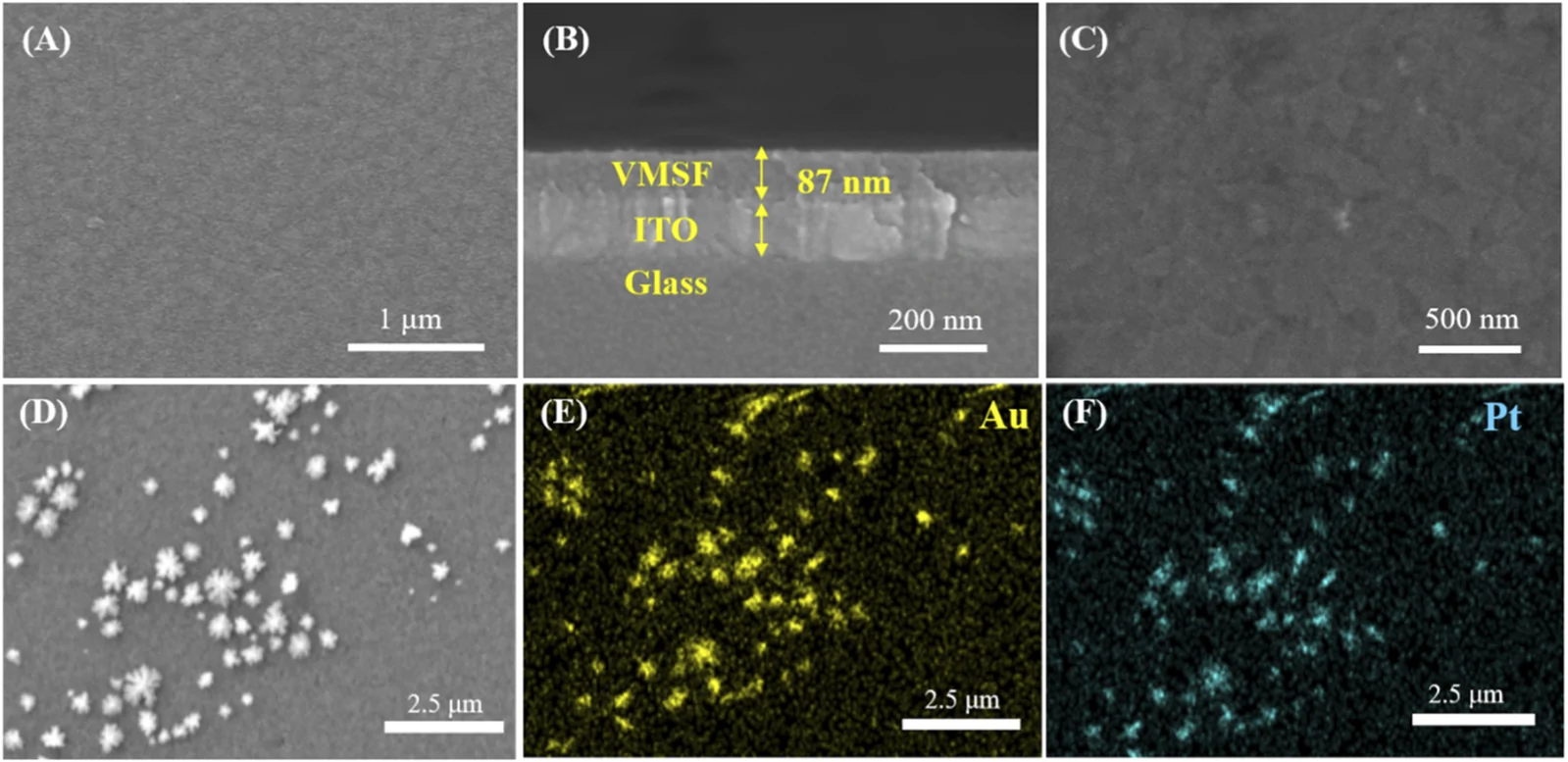
Surface **(A)** and cross-sectional **(B)** SEM image of NH_2_-VMSF/ITO. **(C)** Surface SEM image of AuPt NPs@NH_2_-VMSF/ITO. **(D)** Top-view SEM image of AuPt NPs@NH_2_-VMSF/ITO after removal of NH_2_-VMSF by sodium hydroxide etching, and the corresponding **(E)** Au and **(F)** Pt elemental mapping.

The surface morphology of the electrodes before and after AuPt deposition was examined by scanning electron microscopy (SEM). NH_2_-VMSF/ITO exhibited a smooth and continuous surface without obvious defects, indicating uniform film coverage on the ITO substrate (Figure 2A). Cross-sectional SEM revealed NH_2_-VMSF thickness of ∼87 nm in NH_2_-VMSF/ITO/glass three layered structure (Figure 2B). After AuPt deposition, the film surface remained intact and smooth, and no large particles were observed (Figure 2C). This observation suggested that the metallic deposits were predominantly formed within the silica nanochannels rather than as large aggregates on the external surface.

To investigate the distribution of the metallic deposits, the silica film (NH_2_-VMSF) was etched with sodium hydroxide after AuPt electrodeposition. As shown in Figure 2D, nanoscale deposits were relatively uniformly distributed over the exposed ITO surface. The corresponding elemental maps showed strongly overlapping spatial distributions of Au and Pt (Figures 2E,F), supporting the co-electrodeposition of the two metals. The results confirm the formation of Au/Pt bimetallic deposits in nanochannels.

**FIGURE 3:**
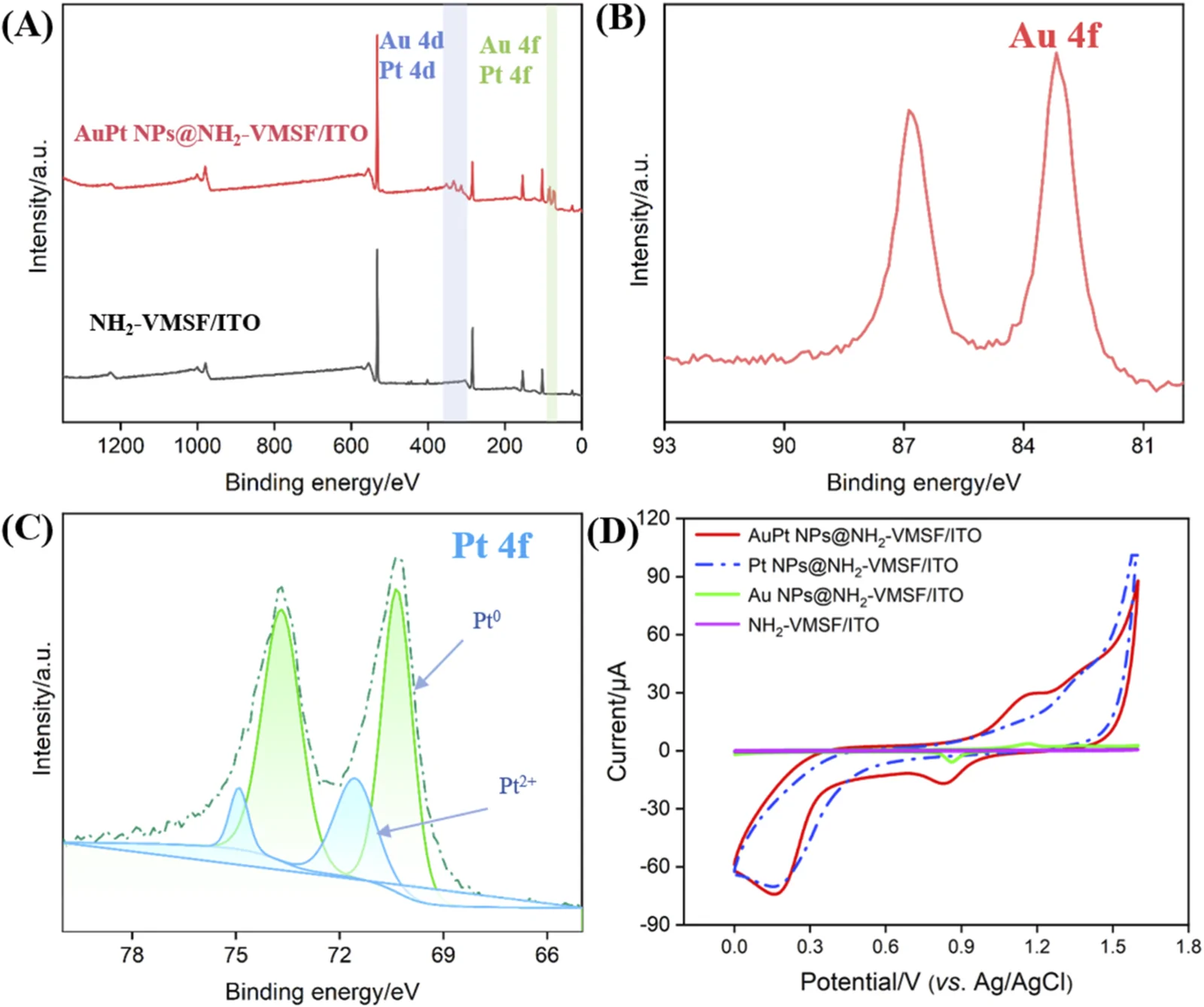
**(A)** XPS survey spectra of NH_2_-VMSF and AuPt NPs@NH_2_-VMSF. **(B, C)** High-resolution spectra of Au 4f **(B)** and Pt 4f **(C)**. **(D)** Cyclic voltammograms of AuPt NPs@NH_2_-VMSF/ITO, Pt NPs@NH_2_-VMSF/ITO, Au NPs@NH_2_-VMSF/ITO, and NH_2_-VMSF/ITO in 0.1 M sulfuric acid.

The X-ray photoelectron spectroscopy (XPS) survey spectrum of NH_2_-VMSF/ITO contained signals of C, O, Si, and N (Figure 3A). After AuPt deposition, distinct Au- and Pt-related signals appeared, confirming successful introduction of both metals. Deconvolution of high-resolution Au 4f spectrum produced peaks at 87.02 eV and 83.31 eV, assigned to Au^0^ 4f _5/2_ and Au^0^ 4f _7/2_ (Zhang et al., 2024), respectively (Figure 3B). In the high-resolution Pt 4f spectrum (Figure 3C), peaks at 70.5 and 74.1 eV were assigned to Pt^0^ 4f _7/2_ and Pt^0^ 4f _5/2_ (Lu et al., 2026b), respectively. Weaker contributions at approximately 71.6 eV and 75.1 eV were attributed to Pt^0^ 4f _7/2_ and Pt^0^ 4f _5/2_ (Figure 3C). The minor oxidized Pt component might arise from partial surface oxidation and charge redistribution in the bimetallic environment. As Au is more electronegative than Pt, electronic redistribution from Pt toward Au might occur when the two metals are in close contact (Zhao et al., 2022). The XPS results therefore support the co-existence of Au and Pt and suggest an electronic interaction between them.

The successful deposition of Au and Pt metals was further examined electrochemically by comparing cyclic voltammograms of different electrodes in 0.1 M sulfuric acid (Figure 3D). Au NPs@NH_2_-VMSF/ITO exhibited characteristic Au-related oxidation and reduction features at ∼1.2 V and 0.89 V, respectively. Pt NPs@NH_2_-VMSF/ITO showed Pt-related electrochemical features at ∼1.5 V and 0.2 V (Tavakkoli et al., 2019). AuPt NPs@NH_2_-VMSF/ITO exhibited electrochemical responses associated with both Au and Pt, further confirming that both metals were deposited at the NH_2_-VMSF modified electrode.

### 3.3 Enhanced ECL by AuPt NPs and optimization of electrodeposition conditions

**FIGURE 4:**
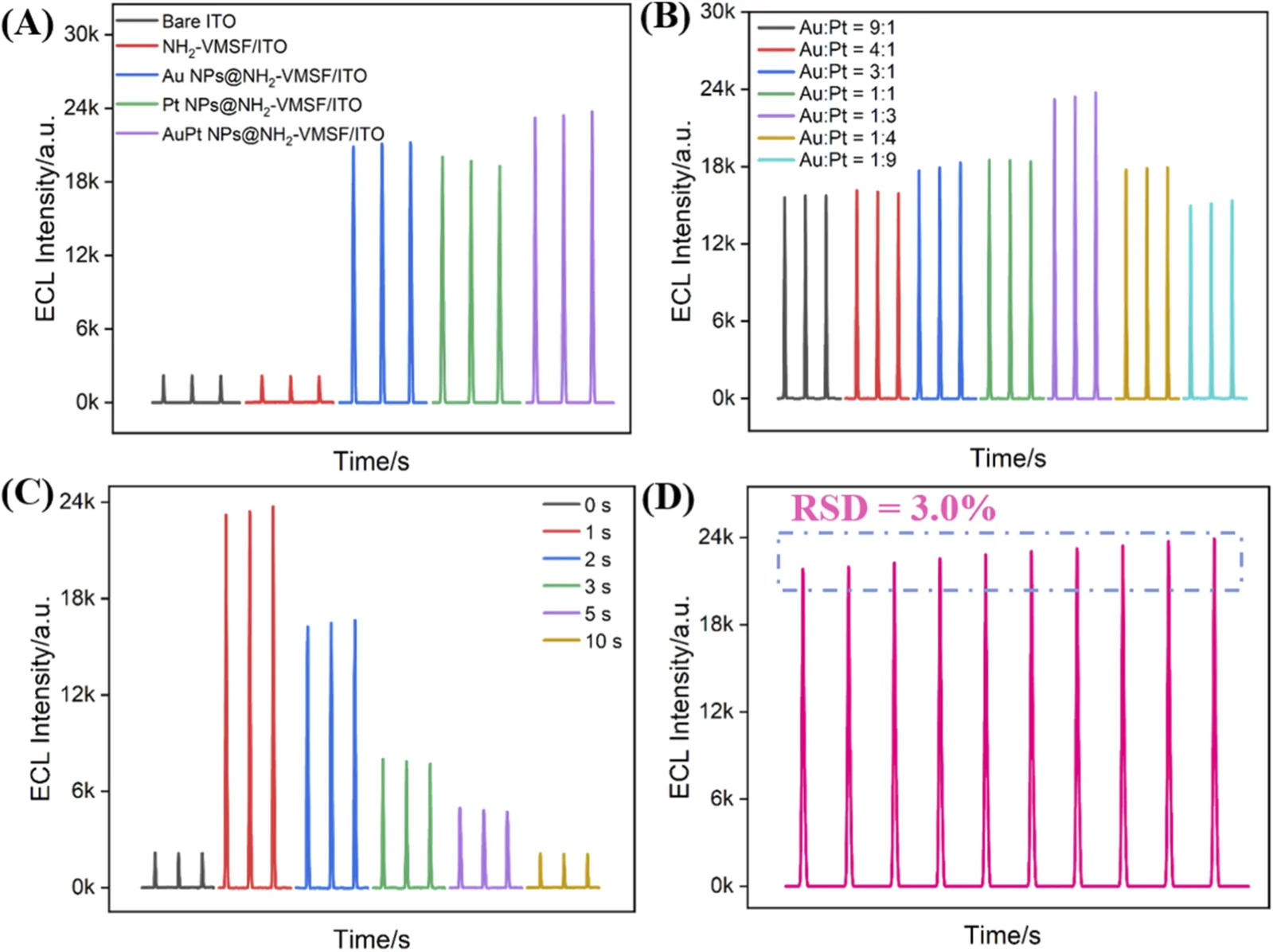
**(A)** ECL responses obtained using different electrodes. **(B)** ECL signals of electrodes obtained using different ratio of precursor solution. **(C)** ECL signals of electrodes obtained using different deposition time. **(D)** ECL signals measured at AuPt NPs@NH_2_-VMSF/ITO electrode for ten consecutive scans.

The catalytic enhancement of luminol-DO ECL by AuPt NPs was evaluated by comparing ECL responses of different electrodes. AuPt NPs@NH_2_-VMSF/ITO generated a significantly higher ECL intensity than bare ITO, NH_2_-VMSF modified electrode or signal metal modified electrodes (Figure 4A). This result indicated that AuPt bimetallic nanostructure provided a stronger ECL enhancement than either single-metal control. As the composition and loading of the AuPt NPs directly affected their catalytic activity, the Au-to-Pt precursor ratio and electrodeposition time were optimized. The maximum ECL intensity was obtained when the molar concentration ratio of Au to Pt precursor in the deposition solution was 1:3 (Figure 4B). ECL intensity was highest at a deposition time of 1 s (Figure 4C). Specifically, a shorter deposition time produced an insufficient number of catalytic sites, whereas prolonged deposition likely caused partial nanochannel blockage or accumulation of metallic deposits near the channel entrances, thereby impeding the transport of luminol. Accordingly, an Au/Pt precursor ratio of 1:3 and an electrodeposition time of 1 s were selected. When the prepared electrode was continuously scanned, the ECL signal exhibited RSD of 3.0%, indicating high stability (Figure 4D).

### 3.4 Mechanism of AuPt-enhanced ECL of luminol-DO

The effective electroactive area of NH_2_-VMSF/ITO or AuPt NPs@NH_2_-VMSF/ITO was calculated using the Randles-Sevcik equation (Fan et al., 2025b). The calculated effective electroactive area was 0.253 cm^2^ for NH_2_-VMSF/ITO and 0.289 cm^2^ for AuPt NPs@NH_2_-VMSF/ITO, respectively. Thus, AuPt deposition produced slight increase in electroactive area.

**FIGURE 5:**
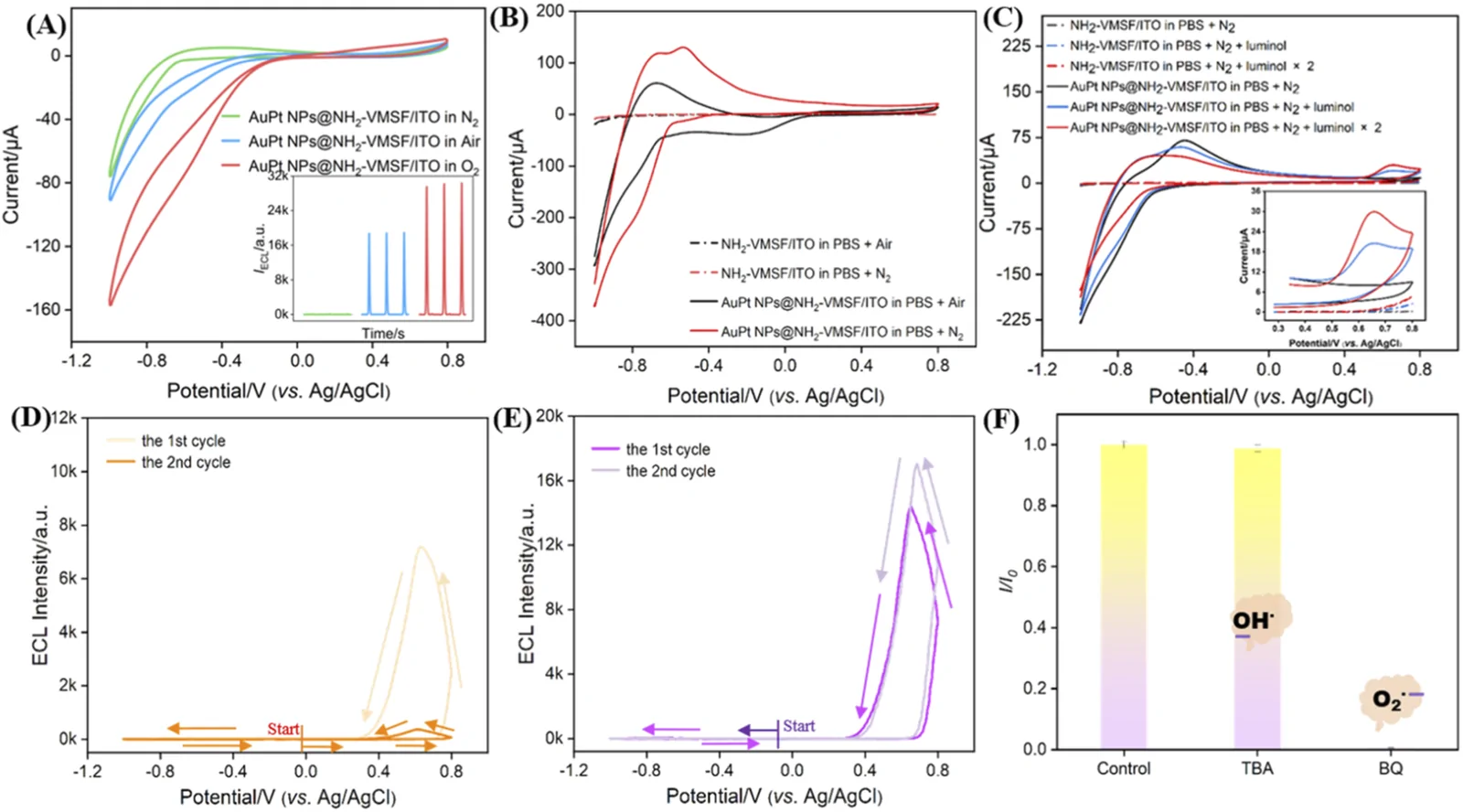
**(A)** Cyclic voltammograms of AuPt NPs@NH_2_-VMSF/ITO under different atmosphere. Inset shows the corresponding ECL responses. **(B)** Cyclic voltammograms of NH_2_-VMSF/ITO and AuPt NPs@NH_2_-VMSF/ITO in PBS under nitrogen or air. **(C)** Cyclic voltammograms of the two electrodes in nitrogen-purged 0.01 M PBS at pH 7.4 containing different luminol concentrations. ECL intensity-potential profiles obtained with **(D)** initially positive-going scan and **(E)** initially negative-going scan. **(F)** ECL response ratio (*I*/*I*_0_) of AuPt NPs@NH_2_-VMSF/ITO before (*I*_0_) or after (*I*) addition of 100 μM TBA or 100 μM BQ.

The involvement of DO in ECL process was investigated by comparing cyclic voltammetry (CV) responses under different gas atmospheres. Figure 5A compared CV and ECL responses of AuPt NPs@NH_2_-VMSF/ITO under nitrogen, air, and oxygen atmospheres. A clear cathodic oxygen-reduction current and a strong ECL signal were observed in the presence of oxygen. Both responses were substantially weaker under nitrogen and became higher under oxygen atmosphere. These observations demonstrate that DO is essential to the strong ECL emission of this system.

In air-equilibrated PB, AuPt NPs@NH_2_-VMSF/ITO exhibited a distinct oxygen-reduction peak near −0.2 V, whereas this peak was remarkedly decreased after nitrogen purging (Figure 5B). These results confirm that AuPt NPs catalyzed the electrochemical reduction of DO. To examine luminol oxidation, CV measurements were performed under nitrogen using different electrode. AuPt NPs@NH_2_-VMSF/ITO produced a larger luminol oxidation response than NH_2_-VMSF/ITO, demonstrating that the bimetallic nanostructure also facilitated luminol oxidation (Figure 5C).

The sequence of luminol oxidation and oxygen reduction was investigated by changing the initial potential-scan direction. When the electrode was initially scanned from 0 to 0.8 V (Figure 5D), luminol was oxidized before ROS had been generated. Consequently, little ECL was observed during the initial positive-going stage. During the subsequent negative-going scan, DO was reduced and ROS were generated. High emission did not occur immediately as the luminol radical intermediate might be short-lived. A strong ECL response appeared when the potential again entered the positive region and newly formed luminol oxidation intermediates reacted with ROS generated during the preceding cathodic scan. In contrast, when the electrode was initially scanned in negative direction (Figure 5E), DO was reduced first. Subsequent scanning toward positive potentials oxidized luminol in the presence of oxygen-derived ROS, allowing ECL response to appear during the first cycle. The signal increased further during the following cycle as the cathodic and anodic reaction sequence was repeated. These results demonstrate that both oxygen reduction and luminol oxidation are required for efficient ECL generation.

Reactive oxygen species (ROS) scavenging experiments were performed to identify DO-derived ROS intermediate. As shown in Figure 5F, the addition of *p*-benzoquinone (BQ), a superoxide scavenger (Li et al., 2022; Li C. et al., 2026; Wu et al., 2026c), strongly suppressed the ECL signal, whereas the addition of tert-butanol (TBA), a hydroxyl-radical scavenger (Xu et al., 2023; Zhao et al., 2025b), produced a much smaller change. These results support the predominant involvement of the superoxide radical anion (O^2•−^) in the ECL process. Thus, the reaction pathway can be expressed as follows:

$${\text{LH}}^{-}-e^{-}\;\overset{\text{AuPt NPs}}{\to }{\;L}^{\cdot -}+H^{+}$$

$$O_{2}+e^{-}\;\overset{\text{AuPt NPs}}{\to }\;O_{2}^{\cdot -}$$

$$L^{\cdot -}+O_{2}^{\cdot -}\;\overset{\text{​}}{\to }{\text{AP}}^{2-*}+N_{2}$$

$${\text{AP}}^{2-*}\to {\text{AP}}^{2-}+h\nu$$

Specifically, luminol (LH) was deprotonated to yield luminol anion (LH^−^), which was then electrochemically oxidized to the luminol anion radical (L^·−^) catalyzed by AuPt NPs. Meanwhile, dissolved oxygen was catalyzed by AuPt NPs to generate superoxide anion radicals (O^2•−^). The luminol anion radical reacted with the superoxide anion radicals to form an excited state (3-aminophthalate, AP^2−*^). The relaxation of the excited state to the ground state (3-aminophthalate, AP^2-^) produced ECL.

### 3.5 Feasibility of stepwise construction of the immunosensor

**FIGURE 6:**
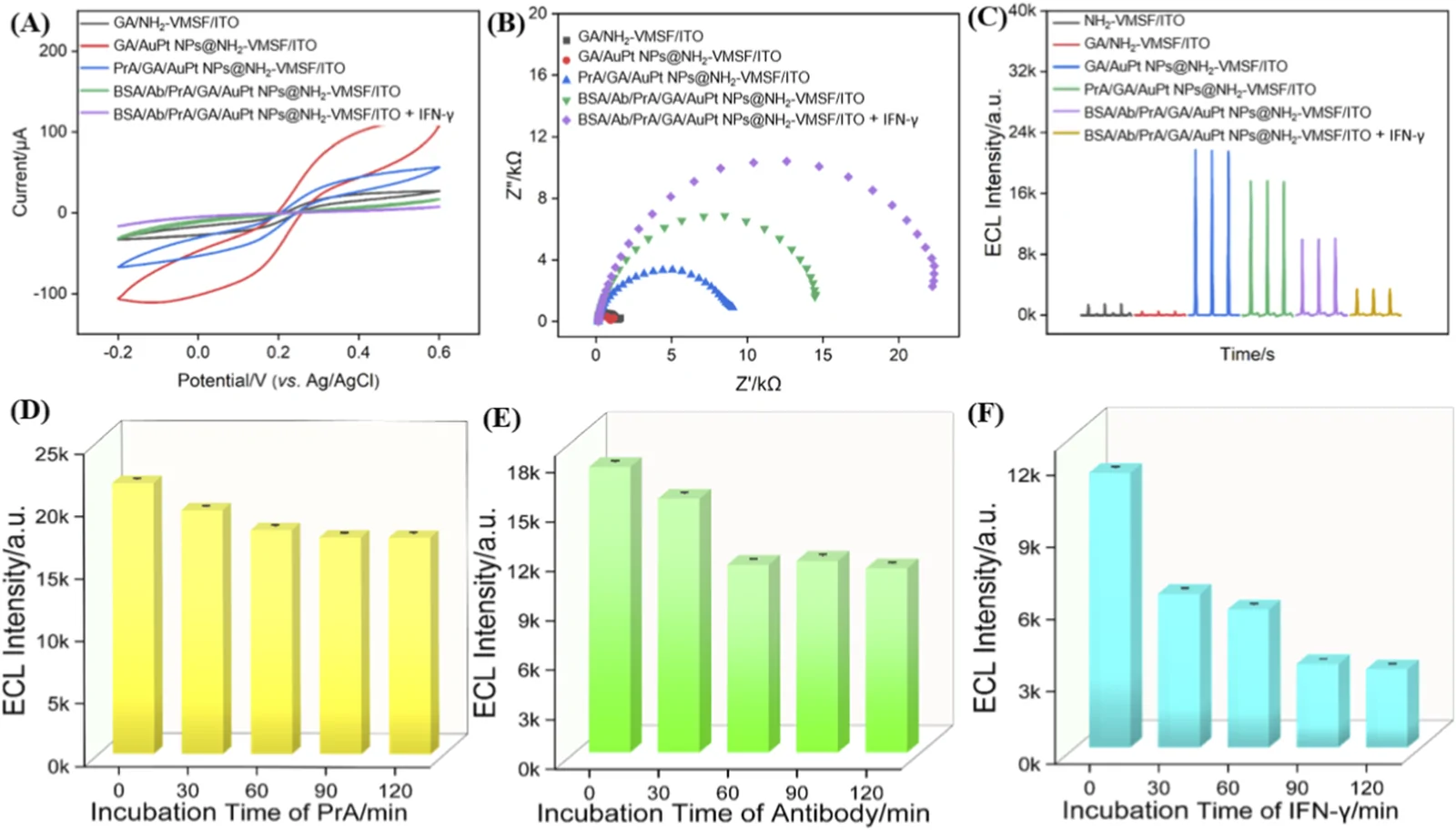
**(A)** Cyclic voltammograms of the electrodes at successive fabrication stages in a solution containing 2.5 mM [Fe(CN)_6_]^3-/4-^. **(B)** EIS plots of successive electrodes in 2.5 mM [Fe(CN)_6_]^3-/4-^ plus 0.1 M KCl. **(C)** Corresponding ECL responses in PBS (0.01 M, pH 7.4) plus 100 μM luminol. Effect of incubation time for **(D)** PrA immobilization, **(E)** Ab immobilization, or **(F)** IFN-γ incubation on ECL response.

CV, electrochemical impedance spectroscopy (EIS), and ECL measurements were used to monitor the stepwise construction of the immunosensor. After GA functionalization, the CV peak current decreased because the GA-modified layer partially covered interfacial transport sites and increased resistance to redox-probe transfer (Figure 6A). After AuPt NPs deposition, GA/AuPt NPs@NH_2_-VMSF/ITO exhibited a higher CV peak current and lower charge-transfer resistance (*R*ct, 738 Ω) than GA/NH_2_-VMSF/ITO (1,160 Ω). These changes indicate that the confined AuPt NPs improved interfacial conductivity and facilitated electron transfer. The sequential immobilization of PrA, anti-IFN-γ antibody, and BSA blocking introduced increasing amounts of electrically insulating biomacromolecules onto the electrode surface, leading to increased *R*ct (9,096 Ω, 14750 Ω, 23071Ω, respectively). At the same time, CV peak current progressively decreased. Following specific capture of IFN-γ, formation of the antigen-antibody complex further increased steric hindrance and resistance to interfacial charge transfer.

The ECL responses exhibited a consistent trend (Figure 6C). AuPt deposition remarkably increased the ECL intensity, whereas the sequential attachment of PrA, anti-IFN-γ antibody, BSA, and IFN-γ progressively decreased it. These results confirm successful construction of the immunosensor *via* stepwise modification and demonstrate feasibility for IFN-γ detection using immunocomplex-induced ECL decrease.

The time for PrA immobilization, Ab immobilization, and IFN-γ incubation were optimized. The ECL response changed with increasing incubation time for PrA immobilization and reached plateau after 60 min (Figure 6D), indicating that the available PrA-coupling sites was saturation. The ECL response associated with Ab immobilization stabilized after Ab incubation time of 60 min (Figure 6E), suggesting sufficient binding of Ab to the PrA-modified interface. The response to IFN-γ reached a stable value after 90 min incubation (Figure 6F), indicating that antigen-antibody recognition had approached equilibrium. Accordingly, incubation times of 60 min for PrA or Ab immobilization, and 90 min for IFN-γ binding were used.

### 3.6 Performance for ECL detection of IFN-γ

**FIGURE 7:**
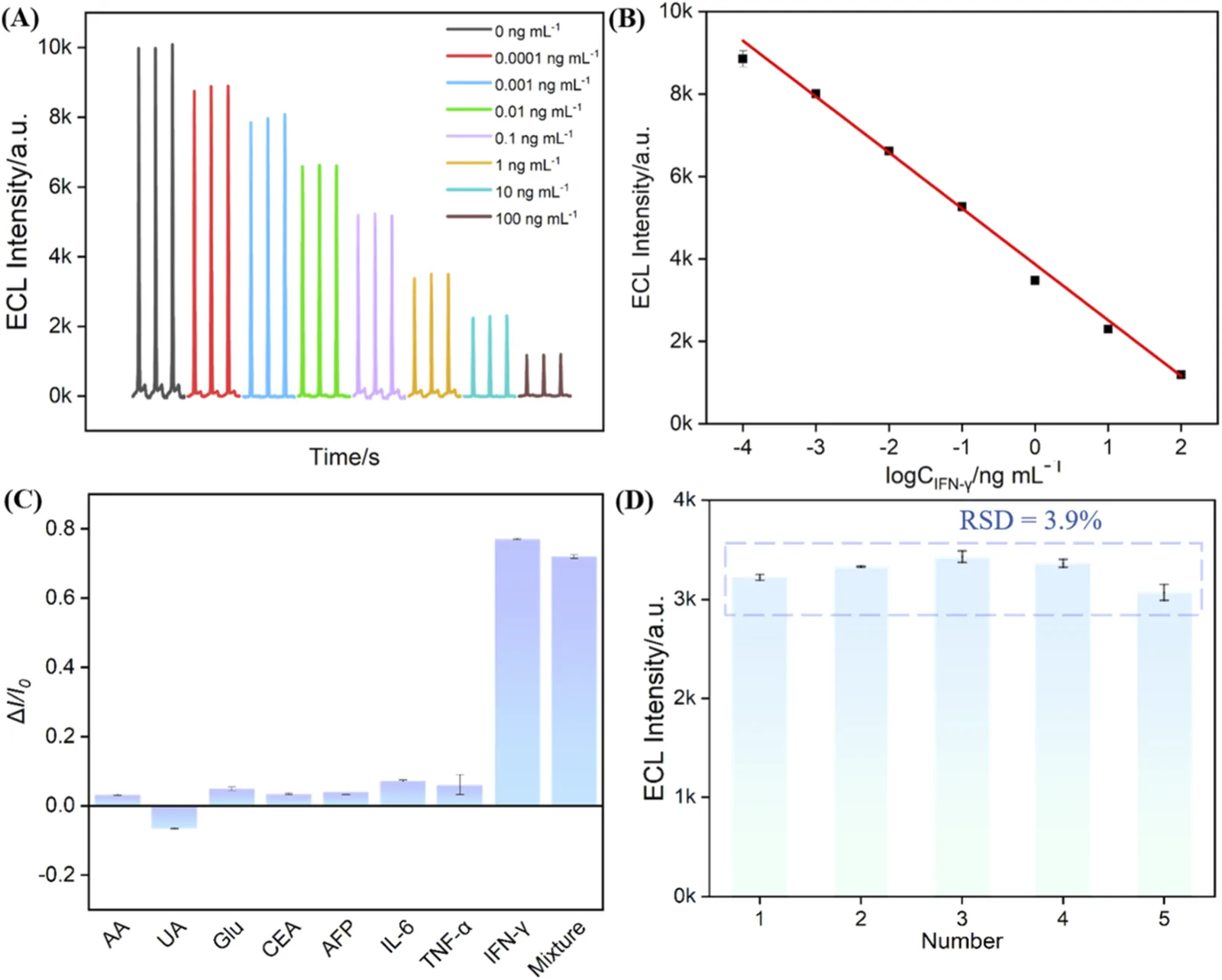
**(A)** ECL responses of the immunosensor after incubation with different concentrations of IFN-γ and **(B)** the corresponding calibration plot. **(C)** Selective responses of the immunosensor to IFN-γ or potential interferent or their mixture. The concentrations of UA, AA, and glucose were 0.1 mM, AFP, CEA, IL-6, and TNF-α were 100 pg mL^−1^. **(D)** ECL responses obtained using five independently prepared immunosensors for detection of 1 ng mL^−1^ IFN-γ.

The analytical performance of the immunosensor was evaluated by incubation with IFN-γ solutions at different concentrations. Then, ECL responses were recorded. The ECL intensity progressively decreased as IFN-γ concentration increased (Figure 7A). The decrease resulted from the formation of increasing amounts of antigen-antibody complexes, which impeded the transport of luminol through the silica nanochannels to the confined AuPt catalytic sites and the underlying electrode. A linear relationship was obtained between the ECL intensity (*I*_ECL_) and the logarithm of the IFN-γ concentration (log*C*_IFN-γ_) ranged from 100 fg mL^−1^–100 ng mL^−1^ with a regression equation of *I*_ECL_ = −1,340 (±45) log*C*_IFN-γ_ (ng mL^−1^) + 3,762 (±101), *R* ^2^ = 0.993 (Figure 7B). Based on a signal-to-noise ratio of 3 (S/N = 3), the limit of detection (LOD) was calculated to be 31 fg mL^−1^. To demonstrate the benefit of oriented immobilization, a control experiment comparing the PrA-mediated oriented immobilization (PrA/Ab, BSA/Ab/PrA/GA/AuPt NPs@NH_2_-VMSF/ITO electrode) with random covalent immobilization (GA/Ab directly without PrA, BSA/Ab/GA/AuPt NPs@NH_2_-VMSF/ITO) was performed (Supplementary Figure S1 in supplementary). The results showed that upon incubation with the same concentration of IFN-γ (1 ng mL^−1^), the sensor with oriented antibodies exhibited a significantly larger decrease in ECL signal (66.8%) compared to the randomly immobilized sensor (51.9%). This might be attributed to the highly accessible Fab regions in the PrA-mediated orientation (Supplementary Material). Supplementary Table S1 in Supplementary Material summarized the IFN-γ detection performance obtained using different sensors. As shown, the fabricated immunosensor displayed low LOD and wide linear detection range. This might be ascribed to the integration of confined AuPt bimetallic catalysis for luminol-DO ECL amplification with PrA-mediated Fc-directed antibody immobilization. This design avoided the need for an externally added H_2_O_2_ co-reactant while improving the accessibility of antibody antigen-binding sites.

The selectivity and resistance to potential interferents were evaluated using other tumor biomarkers including alpha-fetoprotein (AFP), interleukin-6 (IL-6), carcinoembryonic antigen (CEA), tumor necrosis factor-α (TNF-α), or redox compounds including glucose (Glu), uric acid (UA), and ascorbic acid (AA). As shown in Figure 7C, IFN-γ caused remarkable decrease in ECL intensity, whereas the other tested species did not cause significantly change. The immunosensor therefore exhibited selective recognition of IFN-γ and satisfactory resistance to the selected interferents. Reproducibility was evaluated using five independently prepared immunosensors from the same fabrication batch for IFN-γ detection (1 ng mL^−1^). The resulting ECL signals showed RSD of 3.9% (Figure 7D), suggesting good fabrication reproducibility. The storage stability of the immunosensor by investigated by storing it at 4 °C. After 14 days of storage, the sensor retained 91.2% of its initial ECL response to 1 ng mL^−1^ IFN-γ, indicating good storage stability.

### 3.7 Analysis of spiked serum samples

The applicability of the constructed immunosensor was evaluated using spiked FBS as a model complex matrix. FBS was spiked with IFN-γ at different concentrations and diluted 50-fold before detection. As summarized in Table 1, the found concentration (*C*_found_) was in good agreement with the added concentration (*C*_added_). Recoveries ranged from 95.5% to 101.0%, and the RSD was no greater than 1.3%. These results demonstrate that the immunosensor can accurately determine IFN-γ in serum matrix.

**TABLE 1:** Determination of IFN-γ in spiked FBS samples.

Sample^a^	*C*_added_ (ng mL^−1^)	*C*_found_ (ng mL^−1^)	RSD (%, n = 3)	Recovery (%)
FBS	0.0100	0.00955	0.4	95.5
	0.100	0.101	0.2	101
	1.00	0.992	1.3	99.2

## 4 Conclusion

A signal-off ECL immunosensor was developed by integrating nanochannel-confined AuPt bimetallic catalysis with PrA-mediated oriented antibody immobilization. AuPt NPs were electrodeposited within NH_2_-VMSF nanochannels grown on ITO substrate, Au/Pt precursor ratio of 1:3 and deposition time of 1 s provided the strongest ECL enhancement. The AuPt NPs promoted dissolved-oxygen reduction to superoxide radical anions and facilitated luminol oxidation, thereby amplifying luminol-DO ECL response. GA-mediated coupling of PrA enabled Fc-directed immobilization of anti-IFN-γ antibodies to form recognitive interface. IFN-γ capture generated a sterically hindered immunocomplex layer that suppressed the transport of luminol to the confined AuPt catalytic sites and the underlying electrode. The resulting immunosensor provided wide detection range and detection limit. It also exhibited good selectivity, and fabrication reproducibility. This work demonstrates a promising approach for sensitive cytokine determination using dissolved oxygen as a naturally available ECL co-reactant and vertically aligned silica nanochannels as a platform for confined catalytic amplification.

## Data Availability

The raw data supporting the conclusions of this article will be made available by the authors, without undue reservation.
