# Supplementary material for: AuPt bimetallic nanocatalyst-amplified luminol-dissolved oxygen electrochemiluminescence for interferon-γ immunosensing
Source: Front Chem. 2026 Sep 3;14:1942955. doi: 10.3389/fchem.2026.1942955 (PMC13582551; doi:10.3389/fchem.2026.1942955)
Supplement: Supplementary file 1 [file DataSheet1.docx]

Supplementary Material

# 1 Supplementary Experimental section

**1.1. Reagents and materials**

Hexachloroplatinic acid hexahydrate, gold(III) chloride solution, luminol, sodium dihydrogen phosphate hydrate, disodium hydrogen phosphate dodecahydrate, potassium ferricyanide, tetraethyl orthosilicate (TEOS), cetyltrimethylammonium bromide (CTAB), sodium hydroxide, bovine serum albumin (BSA), *p*-benzoquinone (BQ), tert-butanol (TBA), uric acid, ascorbic acid, glucose, glutaraldehyde (GA), and anti-IFN-γ antibody were purchased from Aladdin Biochemical Technology Co., Ltd (Shanghai, China). Sulfuric acid, acetone, absolute ethanol, and concentrated hydrochloric acid were obtained from Shuanglin Reagent Co., Ltd (Hangzhou, China). 3-Aminopropyltriethoxysilane (APTES), potassium hydrogen phthalate, potassium ferrocyanide, and sodium nitrate were purchased from Macklin Biochemical Co., Ltd (Shanghai, China). Carcinoembryonic antigen and alpha-fetoprotein were obtained from Beijing Keyue Zhongkai Biotechnology Co., Ltd. IFN-γ, interleukin-6, and tumor necrosis factor-α were purchased from Sangon Biotech Co., Ltd (Shanghai, China). Protein A was obtained from Oukai Biotechnology Co., Ltd (Nanjing, China). Phosphate buffer solution (PBS, 0.01 M, pH 7.4) was prepared by mixing sodium dihydrogen phosphate and disodium hydrogen phosphate at an appropriate ratio. All reagents were of analytical grade and used without further purification. ITO-coated glass was purchased from Kaiwo Optoelectronic Technology Co., Ltd (Zhuhai, China). The sheet resistance was less than 17 Ω sq−1, and the ITO-layer thickness was 100 ± 20 nm. Before use, the ITO glass was sequentially sonicated in 1 M sodium hydroxide for 1 h, acetone for 30 min, and absolute ethanol for 30 min. It was then sonicated in ultrapure water for 10 min three times and dried under a high-purity nitrogen stream. This treatment exposed surface hydroxyl groups and generated a negatively charged surface. The ITO glass was cut into 0.5 cm × 5 cm strips, and insulating tape was used to define a geometric working area of 0.5 cm × 1 cm.

**1.2. Instrumentation and characterization**

Transmission electron microscopy (TEM) and scanning electron microscopy (SEM) were used to examine the morphology and thickness of NH_2_-VMSF and the morphology of the deposited AuPt NPs. TEM measurements were performed using a Hitachi HT7700 transmission electron microscope, and SEM measurements were conducted using a Zeiss ULTRA 55 scanning electron microscope. For TEM sample preparation, NH_2_-VMSF was carefully scraped from the electrode substrate using a sharp blade and ultrasonically dispersed in absolute ethanol. The resulting suspension was drop-cast onto a carbon-coated copper grid and dried under ambient conditions. To image the AuPt NPs after removing the silica matrix, 50 μL of 2 M sodium hydroxide was drop-cast onto the AuPt NPs@NH_2_-VMSF/ITO electrode to etch the silica film. After 5 min, the electrode was carefully rinsed with ultrapure water to remove residual sodium hydroxide and then dried under ambient conditions before SEM examination. Cyclic voltammetry (CV) and electrochemical impedance spectroscopy (EIS) were performed using a CHI630 electrochemical workstation and a PGSTAT302N Autolab workstation, respectively. X-ray photoelectron spectroscopy (XPS) was conducted using a PHI 5300 electron spectrometer equipped with an Mg Kα radiation source operated at 250 W and 14 kV. ECL measurements were performed using an MPI-E II electrochemiluminescence analyzer. All electrochemical and ECL measurements were conducted using a conventional three-electrode system. An Ag/AgCl electrode and a platinum wire served as the reference and counter electrodes, respectively, while bare or modified ITO was used as the working electrode. Unless otherwise specified, all potentials are reported relative to the Ag/AgCl reference electrode.

**1.3. Preparation of NH_2_-VMSF/ITO**

NH_2_-VMSF/ITO was prepared by an electrochemically assisted self-assembly (EASA) method. Specifically, a precursor solution was first prepared by mixing 20 mL of absolute ethanol, 20 mL of 0.1 M sodium nitrate, 1.585 g of CTAB, 318 μL of APTES, and 2732 μL of TEOS. The mixture was vigorously stirred for 2.5 h. A cleaned ITO electrode was immersed in the precursor solution as the working electrode, and a constant cathodic current density of -1.3 mA cm^-2^ was applied for 10 s. The electrode was immediately removed, thoroughly rinsed with ultrapure water, dried under nitrogen, and aged overnight at 120 °C. The resulting film contained CTAB micelles within its nanochannels (SM@NH_2_-VMSF/ITO). To open the nanochannels, the micelle-filled electrode was immersed in 0.1 M hydrochloric acid in ethanol and gently stirred for 5 min to extract the surfactant template. The electrode was then thoroughly rinsed with ultrapure water and dried under nitrogen, yielding open-channel NH_2_-VMSF/ITO.

**1.4. Preparation of AuPt NPs@NH_2_-VMSF/ITO**

For ECL mechanism study, AuPt NPs were coelectrodeposited within the nanochannels of NH_2_-VMSF using a constant-potential method. The deposition solution contained 0.25 mM gold(III) chloride and 0.75 mM hexachloroplatinic acid in 0.1 M sulfuric acid. Open-channel NH_2_-VMSF/ITO was immersed in this solution, and electrodeposition was performed at -−0.2 V for 1 s. After deposition, the electrode was gently rinsed with ultrapure water to remove residual precursor solution and dried under nitrogen, yielding AuPt NPs@NH_2_-VMSF /ITO. Au NPs@NH_2_-VMSF /ITO and Pt NPs@NH_2_-VMSF/ITO control electrodes were prepared under otherwise identical deposition conditions using the corresponding single-metal precursor solutions.

# 2 Supplementary Figure

**Figure S1.** ECL signal change ratio in presence of IFN-γ (1 ng mL^-1^) obtained on two immunosensor fabricated with or without of PrA. *I*_0_ was the ECL signal obtained before IFN-γ binding.

# 3 Supplementary Table

Table S1 IFN-γ detection performance obtained using different sensors.

| Sensing platform | Method | Linear range  (pg mL^-1^) | LOD  (pg mL^-1^) | Ref. |
| --- | --- | --- | --- | --- |
| HRP-strept/biotin-Ab_2_/IFN-γ/Ab_1_/SPCE | EC | 2.5 ~ 2.0×10^3^ | 1.6 | [1] |
| BSA/IFN-γ Apt/WS_2_NTs/NCPE | EC | 3.1 ~ 0.1×10^3^ | 1.1 | [2] |
| BSA/Ab/In:Ge/Cu@calcein/ITO | PEC | 0.05~ 1.0×10^3^ | 2.4×10^-2^ | [3] |
| MB@Au@CQD-Ab_2_/  IFN-γ/Ab_1_/Au NPs/GO/ITO | ECL | 1.0×10^-2^ ~ 1.0×10^3^ | 1.0×10^-2^ | [4] |
| Ab_2_-CdSQDs//IFN-γ/BSA/Ab_1_/MB@AuNPs/GO-PANI-NF/GCE | ECL | 0.50 ~ 0.5×10^3^ | 3.0×10^-2^ | [5] |
| BSA/Ab/PrA/GA/AuPt NPs@NH_2_-VMSF/ITO | ECL | 0.10~ 1.0×10^5^ | 3.1×10^-3^ | Thiswork |

HRP-strept: peroxidase-labeled strepta-avidin; SPCE: screen printing carbon electrodes; WS_2_ NTs: tungsten disulfide nanotubes; NCPE: nanocomposite paper electrode; In:Ge: indium-germanium doping; Calcein: calcineurin; MB: methylene blue; CQD: carbon quantum dot; GO: graphene oxide; CdSQDs: cadmium sulfide quantum dots; MB@AuNPs/GO-PANI-NF: graphene oxide-polyaniline nanofibers with AuNPs modified magnetic beads; GCE: glassy carbon electrode.

# 4 Supplementary References

[1] Sánchez-Tirado E, González-Cortés A, Yáñez-Sedeño P, Pingarrón J M. *Talanta*, 2020, 211: 120761.

[2] Yeh Y S, Yen Y K, Shanmugam R. *Microchemical Journal*, 2023, 193: 109081.

[3] Ming X, Song C, Yang H, Wang H, Zhang Y. *Chinese Journal of Analytical Chemistry*, 2022, 50(10): 100141.

[4] Zhou B, Zhu M, Qiu Y, Zhang L, Tang Y. *ACS Applied Materials & Interfaces*, 2017, 9(22): 18493.

[5] Zhu M, Tang Y, Wen Q, Zhang Y, Li J. *Microchimica Acta*, 2016, 183(6): 1739.
